# Is There a Reformation Into Identity Achievement for Life After Elite Sport? A Journey of Identity Growth Paradox During Liminal Rites and Identity Moratorium

**DOI:** 10.3389/fpsyg.2021.644839

**Published:** 2021-04-16

**Authors:** Elodie Wendling, Michael Sagas

**Affiliations:** ^1^Department of Kinesiology and Health, Georgia State University, Atlanta, GA, United States; ^2^Department of Sport Management, University of Florida, Gainesville, FL, United States

**Keywords:** identity status paradigm, athletic career transition, identity work, identity growth, liminality

## Abstract

Athletes’ identity development upon retirement from elite sport was examined through a model of self-reformation that integrates and builds on the theoretical underpinnings of identity development and liminality, while advancing seven propositions and supporting conceptual conjectures using findings from research on athletes’ transition out of sport. As some elite athletes lose a salient athletic identity upon retiring from sport, they experience an identity crisis and enter the transition rites feeling in between their former athletic identity and future identity post-sport life, during which a temporary identity moratorium status is needed for identity growth. Given the developmental challenges encountered in moratorium and psychosocial processes necessary to establish a new, fulfilling identity for life after elite sport, we identified key conditions, triggers, and processes that advance how a journey of identity growth paradox experienced during liminality serves as a catalyst toward identity achievement. Elite athletes must be encouraged to persevere in this challenging identity search and delay commitments for as long as it is necessary to achieve identity growth despite experiencing uncomfortable feelings of confusion, void, and ambiguity during the liminal phase. Reforming into an achieved identity for life after elite sport would corroborate the successful navigation of transition, as elite athletes evolved into a synthesized sense of self by cementing, through a negotiated adaptation pathway, constructed identity commitments that will provide new beginnings and meaningful directions to their life after elite sport.

## Introduction

The transition to life after elite competitive sport significantly affects athletes’ well-being upon retirement (Stephan et al., 2003; Holding et al., 2020). Indeed, spending thousands and thousands of hours practicing and competing, elite athletes’ intense focus on sport throughout their lives have made it challenging for them to explore beyond sport (Brewer and Petitpas, 2017; Wendling and Sagas, 2020). Upon retiring from sport, elite athletes may not have many alternatives to build on for structuring a new sense of self. Elite athletes may feel depressed and confused about what should be their next self in their life after sport (Lavallee et al., 2000; Wylleman, 2019). In their attempt to transition into a post-playing identity, former athletes may experience an identity crisis between who they were and who they will be (Kerr and Dacyshyn, 2000). This diametric has warranted the need to investigate the processes underlying identity growth and reformation within the shift from an elite-level playing sport career identity to the next identity they will pursue in life after sport. In their review of athletes’ career transition out of sport studies, Park et al. (2013, p. 38) “found no clear evidence that certain strategies are more effective than others, except for searching for new careers or interests.” The existing literature has advanced several conceptual models of athletes’ transition to life after sport (e.g., Taylor and Ogilvie, 2001; Stambulova, 2003); however, theory building as it relates to an athlete’s identity development upon retirement from elite sport has received only scant attention (viz., Kerr and Dacyshyn, 2000; Lyons et al., 2018). We intend to address this gap in the literature by proposing an integrated model of self-reformation, one that provides a nuanced and in-depth understanding of an athlete’s identity development processes during the transition to life after elite sport.

Our integrative framework is grounded on the anthropological notion of liminality, which is defined as “the experience of being betwixt and between social roles and/or identities” (Ibarra and Obodaru, 2016, p. 53). Liminality has gained popularity in management and organizational sciences when examining the process of a work role change and ensuing role identity transitions (Beech, 2011; Conroy and O’Leary-Kelly, 2014; Hennekam and Bennett, 2016; Ibarra and Obodaru, 2016; Soderlund and Borg, 2017; Gordon et al., 2020). The increased use of this concept coincides with changing career development landscapes, that have increasingly become unstable and uncertain, giving rise to the frequent occurrence of liminal experiences encountered among contemporary workers (Ibarra and Obodaru, 2016). As it relates to the identity transition of an athlete to life after elite sport, we advance this liminality concept as an excellent lens to analyze the experience of being in between an elite-level athlete identity and the forthcoming post-sport identity.

Our model builds on this contention that the transition out of elite sport is a liminal process by also integrating and advancing propositions to describe the complex process of identity reformation during this transition. Based on the view of Soderlund and Borg (2017), identity development is of paramount importance during a liminal phase of transition. To this end, there is a dearth of theoretical integrations of pioneering work of Erikson (1959) around identity development and the Neo-Eriksonian scholarship with the literature on liminality and transition out of sport. Although Ibarra and Obodaru (2016) briefly noted the application of this identity development theory to describe the notion of identity growth during liminality, and Kerr and Dacyshyn (2000) discussed the implications of findings related to athletes’ transition out of sport using work of Erikson (1963), there is a need to offer integrated perspectives of the nuance that plausibly exists in the identity reformation process during the transition to life after elite sport.

**Figure 1:**
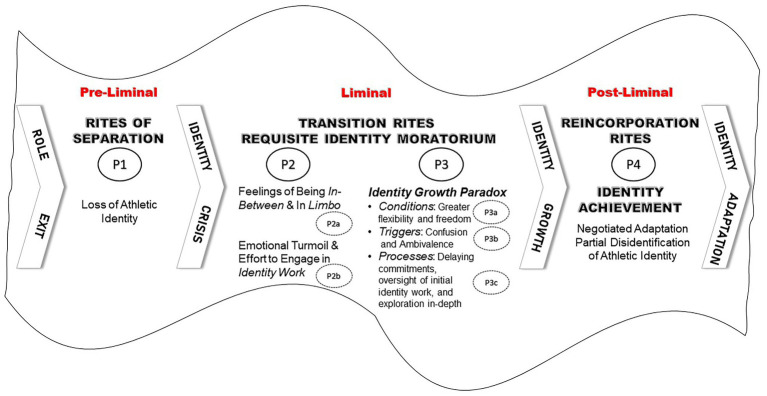
Proposed integrated model of self-reformation during the life after sport transition.

As suggested by Crocetti and Meeus (2015, p. 111), monitoring identity development in various stages of life, such as “when life transitions (marriage, parenthood, and retirement) stimulate the search of new identity structures” would provide important theoretical contributions to the identity literature. Our study advances a unique integrated approach that builds on the tremendously insightful and copious Neo-Eriksonian scholarship, along with the theoretical underpinnings of liminality, in order to better predict several plausible developmental challenges and psychosocial processes necessary for elite athletes to experience identity reformation during their life after sport transition. Thus, we propose an integrated model of self-reformation based on a number of heretofore unconnected but related literatures, including the developmental psychology literature on identity status, the anthropological and management literatures on the concept of liminality, while applying findings from the sport psychology research on athletes’ transition out of sport. Drawing upon these conceptual integrations, we also aim to identify transition related conditions and triggers as well as identity work processes to advance how an identity growth paradox experienced during the transition to life after elite sport serves as a catalyst toward identity achievement. Given that the achievement status has been found to be the best functioning identity status, scoring the highest on a wide array of well-being indices (Kroger and Marcia, 2011; Schwartz et al., 2013), the application of this integrated model of self-reformation could be beneficial to researchers, practitioners, and elite athletes themselves in need to cope with challenging transitions as these proposed heuristic processes could contribute to enhancing elite athletes’ well-being. After providing an overview of the key theoretical concepts of identity development and status, and liminality in the context of the transition to life after elite sport, we introduce our model of self-reformation that integrates these concepts, while advancing seven propositions to support the conceptual model depicted in Figure 1.

## Overview of Theoretical Framework

### Erikson’s Identity Development Theory

As individuals are exposed to new life demands, and earlier identity commitments no longer fit their current life situation, they may fall into a period of disequilibrium caused by an identity crisis. Identity development from an Eriksonian perspective evolves from the absence of a clear, stable, and coherent understanding of who we are and what we seek to pursue in life (i.e., identity confusion) to the presence of such understanding (i.e., identity synthesis; Waterman, 2015). With a fragmented sense of self, individuals enduring identity confusion lack directions to make important decisions in their lives; they feel disorientated and uncertain about deciding what goals to pursue in life as well as what is worth valuing and what to believe (Erikson, 1968). Furthermore, the developmental aspect is indicated by a change in one’s cognitive structure and content of identity (Kroger, 1996). For such a variation to constitute a development, there must be important additions to one’s identity that lead to definite changes in one’s behavior and thinking (Côté, 2015). A movement from a less complex and differentiated identity structure to a more synthesized one denotes a positive identity development (Kroger, 1996). Thus, identity growth is preceded by a period of trial and error and reflection upon which earlier identifications are examined in accordance with current social and cultural contexts and one’s personal values, interests, and talents, in order to discard some of these earlier identity elements and integrate others into a new core identity configuration (Erikson, 1968; Kroger and Marcia, 2011).

### Marcia’s Identity Status Paradigm

Individual differences in how an identity crisis is handled to form a sense of self have been empirically operationalized through identity status paradigm of Marcia (1966). Identity statuses are derived from levels of exploration and commitment such that individuals in: identity achievement have high exploration and commitment; identity foreclosure have low exploration and high commitment; identity moratorium have high exploration and low commitment; and identity diffusion have low exploration and commitment (Marcia et al., 1993). Lacking identity commitments, individuals in moratorium are actively searching for a sense of self, whereas those in diffusion are not engaged in exploration (Marcia et al., 1993). In contrast, individuals in achievement and foreclosure have both established strong identity commitments, but they differ in the amount of exploration they have accomplished prior to resolving their identity crisis.

### Liminality as an Identity Transition Process to Life After Elite Sport

As suggested, the core process of the transition to life after elite sport is conceptualized in our model through liminality, which originated from the major work entitled “Les rites de passage” of the French anthropologist Arnold van Gennep (1960), first published in 1908. In the transition from one identity or role to another (e.g., boy to man), van Gennep (1960) observed that, in all cultures, the rites of passage are divided into three phases: rites of separation (pre-liminal stage), transition rites (liminal stage), and incorporation rites (post-liminal stage). Depicting meaningful ritualistic elements linked with the passage of any transition from the old to the new (Feiler, 2020), this universal sequence is used to frame identity reformation and illustrate an evolving, dynamic, and adaptive transition process to life after sport. Through this lens of work, an athlete transitioning away from an elite-level athlete identity would move along (and at times regress) through the three phases of rites of passage to redefine a new sense of self. As elite athletes start letting go of their athletic identity, they navigate the transition rites, making psychosocial adjustments before moving onto a new meaningful and internalized identity.

While the concept of liminality has received some attention within the realm of sport marketing research (e.g., Green and Chalip, 1998; McCabe, 2006; Chalip, 2008; Rowe, 2008; Bowers, 2011), only a few studies related to the transition out of sport referenced or alluded to the idea that retired athletes experienced a liminal period during this transition (e.g., Kerr and Dacyshyn, 2000; Stephan et al., 2003; Gairdner, 2015). To chart the identity shift athletes in transition experience, Kerr and Dacyshyn (2000) advanced three phases, including retirement, nowhere land, and new beginnings. This sequence was similar to the one provided by work of Bridges (1980) and Feiler (2020) on transition, both of whose research were directly informed and influenced by concept of liminality of van Gennep (1960). In the examination of identity reformation post-elite sport life, existing work provided evidence of existential questioning and deep self-investigation during the transition process (Kerr and Dacyshyn, 2000; Stephan et al., 2003; Gairdner, 2015); findings that effectively describe a liminal experience.

## Rites of Separation and Identity Crisis

In our model of transitioning out of elite sport, the termination of the athlete role, or role exit (Ebaugh, 1988), connotes the separation stage. Indeed, the rites of separation are triggered by a turning point during which the elite athlete role is terminated, from either a normative, voluntary event or a less predictable, involuntary event (Park et al., 2013). Marked by a sense of loss of athletic identity, the separation phase causes significant disruption to athletes’ sense of self (Drahota and Eitzen, 1998; Willard and Lavallee, 2016). Without sport to define their identity, some elite athletes are likely to experience an identity crisis because new life roles may not be available to them in the wake of this change (Holstein et al., 2015; Willard and Lavallee, 2016). Although experiencing an identity crisis may appear to be catastrophic and implies a sense of struggle, from a neo-Eriksonian perspective, this crisis is considered to be a turning point in an individual’s life that would trigger him or her to engage in identity work and exploration to facilitate the formation of a sense of self (Kunnen and Metz, 2015). The source of this crisis originates from a need to change and adjust responses to existential questions such as “who am I?” and “who do I wish to become?.” We also recognize that the level of distress experienced during this transition may vary greatly, with some athletes experiencing this process with little or no distress to others experiencing it as a highly stressful experience (Wylleman, 2019).

Investigating the role exit process, Ebaugh (1988) contended that the challenges of losing a primary source of identity is aggravated if the role exit is unexpected, sudden, involuntary, and irreversible. Unfortunately, these factors are commonly present in elite athletes’ exit from sport, since the end of their career is frequently out of their control, as in the case of a career-ending injury or being released from a team (Holstein et al., 2015; Wylleman, 2019). For athletes who did not plan prior to retiring from elite sport, a role exit that is unanticipated and obligatory in nature would intensify the difficulties of leaving sport (Butt and Molnar, 2009; Holstein et al., 2015).

The separation process can be challenging for some athletes who may be experiencing feelings similar to withdrawal symptoms. As described by Drahota and Eitzen (1998, p. 263), the role exit of athletes is “a difficult time for [them] because they lose what has been the focus of their being for most of their lives, the primary source of their identities, the physical prowess, the adulation bordering on worship from others, the money and the prerequisites of fame, the camaraderie with teammates, and the intense ‘highs’ of competition.” Athletes’ physical, emotional, financial, chemical, and mental attachment to the game enhances the difficulty to disengage from their athletic life and establish a new sense of self (Drahota and Eitzen, 1998). Elite athletes in the process of forming a new identity may, however, build on their past high-performance sport experience and incorporate some form of their athlete identity in this new sense self of self, a notion that will be further explained in the final proposition of the model. While athletes struggle to disentangle themselves from the past, social expectations add to the tension of letting go their elite-level athlete identity, as people often continue to treat athletes based on whom they used to be (Drahota and Eitzen, 1998). Regardless of the magnitude and intensity of the role exit process, the loss of the athletic identity causes elite athletes to experience a separation from a salient sense of self that leaves them with no other choice but to begin letting go of their athlete self.

The separation phase does not have to coincide with the official departure of an athlete, as he or she can be going through the vacuum stage of the role exit process prior to retiring from sport. According to Ebaugh (1988, p. 23), “role exit is process that occurs over time.” As explained by Ibarra and Obodaru (2016), professional workers who are dissatisfied with their job, may contemplate a career change and undergo a liminal experience long before they leave their employment (if they leave at all). When some elite athletes can anticipate the end of their sport career, they may be propelled into liminality, while they are still active athletes. As demonstrated by the contemporary descriptions of liminal experiences, it has been challenging for researchers or elite athletes themselves to clearly identify when the transition process starts (Knights et al., 2016). Regardless of the idiosyncratic timing of this process, athletes must be able to let go of their former self as an elite-level athlete to be ready to move onto the liminal stage. Hence, we developed the following proposition in relation to the pre-liminal stage:

*Proposition 1*: As a turning point, role exit from a high-performance sport career or the anticipation of it is postulated to trigger the separation stage of transition, a stage that is marked by a loss of a salient athletic identity (or upcoming one), which is expected to prompt an identity crisis.

Given the importance of identity reformation once individuals undergo an identity crisis, concepts of identity status and growth must be integrated in the core stage of this liminality model, which, in addition to describing the liminal stage, will be the focus of our next section.

## Transition Rites and Requisite Identity Moratorium

The separation stage leads to the start of an indeterminate state that is ambiguous for athletes, as they are in between letting go of the athlete identity and moving on to a new identity. Athletes in this stage can be referred to as “liminars.” Originating from the Latin term limen, which means a “threshold,” liminality refers to the transition period in which individuals are “no longer what they were, nor yet what they will” (Rowe, 2008, p. 128). Drawing on a work of van Gennep (1960), Turner (1967) extended the conceptualization of liminality, viewing the liminal period as an “interstructural situation” (Turner, 1967, p. 93), during which individuals are in betwixt and between conditions. During this phase, liminars have a few or none of the characteristics of the old and new states. Ibarra and Obodaru (2016, p. 49) denotes an “identity limbo” to illustrate how liminars are suspended in between the past and future positions. The idea of “no man’s land” is echoed in work of Turner (1967, p. 96), being “neither one thing nor another.”

Applied to the life after elite sport transition, liminality would refer to a period in which athletes feel “in-between” the athlete identity and the next identity they decide to pursue in their post-elite sport life. Evidences of Turner’s conceptualization of liminality were observed in a few sport career termination studies. For instance, Stephan et al. (2003) explained how their athletes were in a liminal position, feeling in between the status of athlete and full-time employee. Similarly, Kerr and Dacyshyn (2000) reported that their former athletes experienced feelings of being suspended in between two worlds. They were not completely moved on from their former identity as an elite competitive athlete and not yet found or fully assimilated their post-sport identity. Based on the above literature, the following proposition is advanced to support the initiation of the transition rite of liminality for athletes:

*Proposition 2a*: Elite athletes enter the liminal stage of a transition when they are experiencing feelings of being in limbo and being suspended in between the loss of athletic self and the absence of future directions.

In addition to losing their athletic self and feeling in between two worlds, athletes may feel directionless as they embark on a search for a new identity (Lavallee and Robinson, 2007). The notion that transitioning out of elite sport can leave athletes confused about what should be their next self was evidenced multiple times in the literature (Grove et al., 1997; Kerr and Dacyshyn, 2000; Lavallee and Robinson, 2007; Warriner and Lavallee, 2008; Douglas and Carless, 2009; Park et al., 2013). Once the playing days are over, athletic identity loss triggers a disruption to athletes’ sense of self that is characterized by instability during which athletes may feel an incomplete sense of self. In addition, this disruption and instability may cause athletes to experience a loss of meaning in life and a struggle to fill the void as they feel uncertain about what their next chapter in life should be (Grove et al., 1997; Drahota and Eitzen, 1998; Stephan et al., 2003; Cavallerio et al., 2017). For instance, Warriner and Lavallee (2008, p. 306) highlighted two major themes pertaining to the experiences of sport retirement, which were “loss and turmoil” and “identity confusion,” with participants describing their disengagement from elite sport as “profoundly traumatic.” Emerging theme of nowhere land of Kerr and Dacyshyn (2000) closely resembles liminality, during which former athletes felt disoriented and confused, losing meaning and control in their lives due to the uncertainty of their future endeavors. Thus, retired athletes have reported to experience a liminal state during the transition to life after sport, given that feelings that are commonly observed during liminality include confusion, uncertainty, void, doubts, anxiety, ambiguity, and disorientation (Kerr and Dacyshyn, 2000; Cavallerio et al., 2017).

When facing an identity crisis as posited by Erikson (1968) and experienced during a liminal period such as transitioning to life after elite sport, individuals must make choices in terms of what to explore and which commitments to make, decisions that are all made while being in moratorium (Marcia, 1966). Given that positive identity development is preceded by a period of active exploration, it appears that the moratorium status is a requisite with respect to identity formation, and more importantly identity growth (Marcia, 1966). As explained by Marcia (1993), individuals in moratorium are going through a provisional but necessary transition to experience identity growth. Thus, we contend that moratorium is most akin to liminality as liminars capitalize on this liminal state to engage in identity work and achieve a synthesized sense of self.

During this identity search and the process of constructing an identity, Marcia (1993, p. 8) described individuals in moratorium as “trapeze performers, holding on to the bar of the past, while swinging toward that of the future, often with much of the vacillation, fear, intensity, and excitement connoted by the circus image.” This illustration closely resembles the concept of liminality. Thus, the liminal phase, during which identity crisis is experienced, would require retired athletes to go through a moratorium period to resolve their identity concerns and reach an evolving and constructed sense of self for their post-sport lives. Given the sole path to identity growth calls for a temporary moratorium period, the following proposition was developed to further describe the liminal stage of transition in life after elite sport and integrate the identity status paradigm to advance the requisite of identity growth during liminality:

*Proposition 2b*: The liminal stage is marked with psychological challenges and emotional turmoil as elite athletes are expected to make an effort to engage in identity work, such that a requisite identity moratorium is postulated to be the identity status most akin to the liminal stage in order to foster identity growth.

However, in the attempt to forge an identity, individuals in moratorium may face difficulties to identify a direction in their lives. They may appear to be worried and struggling to define themselves since they have not found answers to their identity-related questions (Crocetti et al., 2008; Meeus et al., 2010). This period of confusion is aggravated by athletes’ insufficient knowledge of who they are and what their interests, abilities, and values are outside of sport (Cummins and O’Boyle, 2015). The dearth of opportunities to explore interests outside of athletics and develop non-athletic competences during their playing days can cause them to lack confidence in areas outside of sport, which can also discourage them to try non-sport activities and new situations (Brewer et al., 2000). Due to the struggle and emotional trouble that come with being in moratorium (Côté and Schwartz, 2002; Meeus et al., 2010), the resolution of an identity crisis through the exploration of various identity alternatives is not always assured (Côté, 2006). Yet, the transition literature (viz., Dutton et al., 2010; Conroy and O’Leary-Kelly, 2014) demonstrated that a prerequisite to crossing the threshold out of liminality and into a reincorporated state consisted of individuals experiencing and documenting identity growth. Therefore, in the forthcoming part of transition rites and requisite identity moratorium, we advance three propositions related to transition conditions, triggers, and processes through the conceptualization of an identity growth paradox embedded within the liminal stage.

### Identity Growth Paradox as a Catalyst Toward Achievement in Life After Elite Sport Transition

The path to traverse liminality and grow into an achieved identity is not a linear and straightforward process (Gordon et al., 2020); hence, we explain, in turn, key conditions, triggers, and processes underlying this path to demonstrate the identity growth paradox of the transition to life after sport that is critical to forge a constructed identity for once athletes’ elite-level sport career ends.

Typically, the conditions surrounding athletes’ transition are conducive to identity growth, as they provide great autonomy to athletes, enabling them to be creative, innovative, and agentic in crafting their post-liminal identity. Drawing upon the work of Ibarra and Obodaru (2016) around contemporary liminal experiences, we contend that the uncertainty of the duration and outcome of the life after sport transition, and the idiosyncratic aspect of developing an identity and making sense of this transitioning passage afford enough flexibility to stimulate innovation and reduce the need for elite athletes in transition to conform to external pressures.

The duration of being “in between” the athlete identity and the next identity is not predetermined and can be enduring for as long as it is necessary to discover a new self and/or cope with turbulent emotions. Depending on the reasons for retiring from sport and the amount of preparation prior to retiring, athletes may remain suspended between the old and new identities for an indeterminate period of time (Park et al., 2013). Similarly, a post-liminal identity may be unknown for many athletes at the onset of their transition. The uncertainty of identifying meaningful identity commitments once they leave their athletic career combined with the lack of a guaranteed positive identity development pathway demonstrate the difficulties of navigating this transition; conditions that also paradoxically offer favorable conditions to foster identity growth. Furthermore, the liminal experience of athletes is mostly self-guided, as the separation from their teammates and coaches upon retirement may require them to deal with the challenges of the transition on their own (Coakley, 1983; Lavallee et al., 1997). Although the lack of formal and collective structures would require athletes in transition to build their own support group to guide them through this process, this individualistic and flexible approach to social guidance may stimulate agency, independence, and creativity in crafting their post-liminal identity. The self-directed process of identity work effort and the idiosyncratic approach to meaning making during this transition are evidences of the lack of prescribed steps and legitimate narratives that can be used by transitioning athletes to help them build a new identity and make sense of their transition. Given that all these conditions provide greater potential for identity growth, the following proposition was formulated to support our conceptual model:

*Proposition 3a*: The transition to life after elite sport is posited to offer conditions that afford athletes to experience greater degrees of freedom in constructing their next self, and the resulting enhanced imagination, innovation, creativity, and agency would facilitate positive identity development once they end their elite-level sport career.

In addition to providing grounds to support identity growth, these conditions are auspicious to enhance feelings of confusion and ambivalent emotions. Because these conditions can aggravate the turbulence inherent to being in limbo, they also illustrate the identity growth paradox underlying this transition process. We further contend that, although uncomfortable to endure, these feelings and emotions serve as triggers that can catalyze individuals to progress toward an identity synthesis. Undergoing identity crisis can in fact prompt individuals to search for different possibilities and experiment with various identity alternatives prior to committing to one, which are necessary steps to achieve a stable and unique identity (Erikson, 1968). As previously proposed, athletic identity loss may cause an identity crisis, which would therefore incite former athletes to search for meaning in life (Stephan et al., 2003) and explore neglected identities (Lally, 2007). During the in between phase, to move from disorientation to reorientation, athletes reported to search for meaning and spend time contemplating on their past athletic experience (Kerr and Dacyshyn, 2000), which are all conducive steps for identity growth.

Likewise, the confusion athletes experience during this transition is viewed as a trigger for identity work and existential inquiry that would catalyze identity growth (Gairdner, 2015). In this struggle for self-reformation, feelings of disorientation elicited a period of existential concerns during which athletes pondered questions such as “Who am I?” and “What is next?” (Kerr and Dacyshyn, 2000, p. 122). They expressed feelings of void that fostered the need to find new activities that would bring similar satisfaction and fulfillment as their athletic participation once did. Lavallee et al. (2000) also explained that feelings of anxiety can serve as a spur for personal growth by encouraging athletes to develop a deeper self-understanding and identify new configurations of meaning for their life after elite sport. Therefore, the loss of the athletic identity and ensuing identity confusion can serve as catalysts for identity growth by inciting athletes to search for different possibilities and meaning in life that would be necessary to craft a new sense of self and adapt to their new life.

Furthermore, the awakening of ambivalence experienced during this liminal phase is likely to prompt individuals to engage in identity work and craft a new meaningful and evolving sense of self (Ibarra and Obodaru, 2016). In fact, feelings of ambivalence were found in organizational studies to promote identity growth (Maitlis, 2009; Dutton et al., 2010; Pratt and Pradies, 2011; Ashforth et al., 2014; Conroy and O’Leary-Kelly, 2014). The sport psychology literature on athletes’ retirement from sport detected traces of ambivalence experienced during transition on numerous occasions. For instance, in study of Kerr and Dacyshyn (2000, p. 122), former athletes reported that they experienced “both positive and negative emotions throughout their transitions.” Similarly, several studies underlined mixed feelings and contradictory sentiments with participants expressing a sense of relief from the demands of their athletic career and freedom from strict schedules they had to follow, while also experiencing a sense of loss, sadness, and fear from leaving their athletic career (Coakley, 1983; Sinclair and Orlick, 1993; Kerr and Dacyshyn, 2000; Stier, 2007; Gairdner, 2015; Willard and Lavallee, 2016; Cavallerio et al., 2017). Indeed, the sudden unstructured life and independence can cause athletes to feel lost as they no longer need to abide by a well-regimented life. Thus, positive sentiments of relief and freedom can be accompanied with feelings of uncertainty caused by identity loss as former athletes attempt to find a new path to pursue in their life (Kerr and Dacyshyn, 2000).

Taken together, these studies demonstrated the presence of oscillations between positive and negative feelings that illustrate this unstable state of transition, a journey of paradox, typical of such a form of liminal experience as transitioning to life after sport. The ambivalence of this transition can foster identity crafting and in turn help transitioning athletes experience identity growth. Thus, the ensuing proposition was developed in support of this paradox:

*Proposition 3b*: When athletes enter the in between state feeling confused and/or having ambivalent emotions, although uncomfortable, they are more likely to experience identity growth because these feelings and emotions serve as a catalyst for identity work and existential inquiry necessary to craft a new, meaningful sense of self in life after sport.

While confusion and ambivalence can trigger identity growth, these feelings and emotions may be uneasy for some athletes to undergo, leaving them at risks of shortening liminality by prematurely settling into a “serviceable, secure identity” (Kroger, 1996, p. 209). Long-lasting challenges and stressful in between phases may lead them to avoid delaying commitments by falling into conforming with a conferred sense of self instead of taking the time to explore various options to reform a constructed sense of self. Foreclosing on options too early to avoid uncomfortable feelings of searching for suitable alternatives would be viewed as a maladaptive adjustment to life after sport from an identity development perspective.

Making sudden choices by defaulting back into familiar territories to attempt to end challenging emotions experienced in moratorium would seemingly alleviate confusion and solve identity concerns. Athletes’ inability to make progress toward an achieved identity, however, would squander an opportunity to capitalize on a fruitful transition. In fact, a successful resolution of identity concerns requires individuals to capitalize on this interlude by exploring alternatives (Côté, 2006). A conforming and avoiding approach to forming a sense of self would therefore not be recommended to achieve a coherent and mature identity. Although identity commitments provide foundational directions in an individual’s life, we assert that athletes in transition to life after sport must postpone establishing firm commitments until sufficient identity work has been accomplished to resolve their identity concerns and reach identity achievement.

However, this exploratory period may be lengthy and confusing, particularly for retired athletes who never had to search for a sense of self outside of sport (Grove et al., 1997). While some athletes are able to capitalize on the breadth of opportunities, the availability of various choices can make this developmental task cumbersome and overwhelming to accomplish for others. These difficulties may preclude athletes from adopting an extensive exploration and deliberation of potential alternatives to solve their identity concerns. In addition, athletes are at risk to fall into ruminating the purpose of their existence as they may have not explored enough outside their athletic boundaries and have limited possible identities to fall onto upon their retirement from sport. Losing a dominant athlete self, they may feel as if their life is meaningless without sport, which may lead them to experience existential ruminations (Taylor and Ogilvie, 2001). As well-described by Kroger and Marcia (2011, p. 35), individuals can “appear to be drowning in their struggles to swim against the tide of earlier authority-based identifications. Rather than explorers, they become ruminators, perpetually mired in what seem to be insoluble dilemmas.” When moratorium becomes a permanent status, individuals are stuck in an incessant search for a new self, in which case, athletes end up stagnating in their identity development. Perpetual ruminations and internal conflicts paralyze their abilities to make decisions, leading to maladaptive reactions that would preclude athletes from solving their identity concerns.

However, the source of distress and discomfort may stem from the initial stages of identity search, especially when individuals explore various alternatives in breadth (Crocetti et al., 2008; Meeus et al., 2010; Wendling and Sagas, 2019). It is worthy of note that requisite identity work processes to resolve the identity crisis and underlying the moratorium status include exploring both in breadth and in-depth (Luyckx et al., 2008; Wendling and Sagas, 2019). During the early stages of exploration, individuals are considering broadly, various alternatives by gathering general information and experimenting with options whereas during latter stages of exploration, individuals are implementing and evaluating deeply, fewer options. Once individuals have been able to narrow down their choices they were exploring broadly, they can investigate more deeply these choices to acquire a more refined and specific understanding of these potential commitments. While implementing these initial commitments, individuals are monitoring the viability of these chosen options to either strengthen them further or reconsider them if they are not satisfied with their in-depth assessment (Waterman, 2015).

Exploring in-depth from a more stable and secure base of holding initial commitments, athletes who are implementing and evaluating these choices were found to exhibit better functioning than those who wander in diverse paths and lack directions in their lives because they have not been able to focus on fewer options (Crocetti et al., 2008; Wendling and Sagas, 2019). There is therefore a need to take into consideration how advanced an individual is in the exploratory process, given that the developmental challenges associated with identity work may be alleviated once athletes are no longer considering various identity alternatives. A more advanced stage in the identity work process may indeed lead to a better adaptation in finding new life directions. Thus, we suggest that it is critical to offer support in the early phase of exploration in order to help elite athletes select a few alternatives that they can investigate in depth. As the moratorium period calls for a purposeful, organized, and systematic engagement in exploratory activities, athletes with a fragmented sense of self would benefit from an environment that is supportive of providing structure and oversight for their identity work effort.

These processes of identity growth underline the paradox that surrounds positive identity development during the transition to life after elite sport. Thus, the last proposition related to this paradox and embedded within the liminal stage is as follows:

*Proposition 3c*: In addition to avoiding prematurely settling into a conferred sense of self, athletes exploring in-depth are more likely to experience identity growth than those exploring in-breadth, the latter of which requires support and oversight to ensure that athletes are not falling into perpetual ruminations and that initial identity work leads them to narrow down their options for an eventual formation of a synthesized identity.

In the quest for a new identity, elite athletes must find a way to reprioritize their interests and activities, and reorient their expectations to accommodate changes, shifting their focus from athletic goals and competencies to new ones (Stephan et al., 2003), which is the focus of the last section.

## Reincorporation Rites and Identity Achievement

A progressive identity shift toward a synthesized identity is depicted *via* the formation of an achieved identity, which characterizes the ideal outcome of liminality to experience identity growth during the transition to life after elite sport. In identity achievement, individuals established a constructed sense of self whereas those in foreclosure acquired a conferred sense of self due to their lack of exploration (Marcia et al., 1993). According to Schwartz et al. (2011), foreclosed individuals reported significantly lower scores on meaning and purpose in life, and internal locus of control compared to achievers. Longitudinal work also showed that achievers exhibited better functioning and developmental outcomes than those foreclosed (Schwartz, 2007). Thus, given the importance of self-direction, adaptation, and identity work in the formation of a synthesized identity, identity foreclosure is viewed as a less mature status than identity achievement, the latter of which is considered the most optimal identity status and comes closest to Erikson’s (1968) definition of identity synthesis (Marcia et al., 1993).

Similarly, the contemporary work role transition literature has identified a negotiated adaptation pathway in which individuals adapt and reconfigure their identities during role changes (Wittman, 2019). According to Wittman (2019, p. 728), in this pathway, “people refurbish, recombine, and amalgamate their identity structures to craft new identities to match their new roles.” Thus, this negotiated adaptation would serve as the positive identity development outcome for the life after sport identity transition. Similarly, Ibarra (1999, p. 765) also viewed this path as one in which “people adapt aspects of their identity to accommodate role demands and modify role definitions to preserve and enact valued aspects of their identity.”

Scant empirical evidence exists in the athlete transition literature that effectively describes the rite of reincorporation. One notable exception is offered by Drahota and Eitzen (1998) in their analysis of the shift to a former athlete identity, in which they observed that many years has passed after the athletic career ended before athletes could accept that they were no longer athletes. As explained by Kroger (1996), individuals may experience overwhelming separation anxiety from having to reconsider lingering values from a previous identity. Some athletes may in fact have a difficult time to traverse this transition and disassociate with the athlete identity because they continue to hold on tenaciously to the athlete self that has been so pivotal in their lives (Drahota and Eitzen, 1998; Stier, 2007). However, the search and formation of a new identity can be facilitated by building on core athletic skills and beliefs. As described by Wittman (2019) in the work role context, athletes who reincorporate through a negotiated adaptation would not completely *dis*identify with all aspects of their former identity. Although athletes must be able to come to terms with their former athlete self to adapt to a new identity (Drahota and Eitzen, 1998), they can still retain identity elements (e.g., discipline, confidence, commitment, competition, excitement, and community) that could be connected with new identity elements to create new identity subclusters (e.g., high-public profile jobs, entrepreneurial projects, and high-risk, high-reward businesses, and sport-related education and occupations; Holstein et al., 2015; Lupo et al., 2018; Wittman, 2019; Mateu et al., 2020).

Athletes who are transitioning to a new identity that is congruent to certain aspects of their previous athlete identity could effectively “negotiate” which of the identity elements from their athletic life could be reconfigured and retained and which could be *dis*identified. While a detachment from the former identity must occur to embrace the new one, finding closure with the athletic identity does not mean to completely discard their athletic experience. Taking into account their past and combining it with the new identity, as supported in the Eriksonian identity literature, would enable athletes to make sense of their athletic career (Grove et al., 1998) and would serve as a form of continuity in their identity development in spite of the rupture (Erikson, 1968). In the rite of reincorporation, we would therefore contend that athletes successfully navigate the rite of transition and evolve into coherent sense of self by cementing a new identity through a negotiated adaptation pathway (Ibarra, 1999; Ashforth, 2001; Ibarra and Barbulescu, 2017). As a result, to enhance the prospect for well-being post-elite sport life, we developed this final proposition:

*Proposition 4*: Identity achievement is suggested to be the identity status most akin to the reincorporation stage, which is posited to mark the consummation of the transition from athlete to a constructed sense of self through a negotiated adaptation pathway.

While adaptative adjustments post-elite sport life is indicated by an achieved identity, the reincorporation rite ultimately leads athletes to return to a more stable state during which they are able to redefine a salient self in their life after elite sport that leads to new beginnings.

## Discussion

The developmental challenges and psychosocial processes necessary when athletes transition to life after elite sport were depicted through this integrated model of self-reformation, in such a way that the occurrence of liminal experiences during this transition was deemed as an opportunity for identity reformation that would result in the discovery of a new, meaningful identity for athlete’s life after elite sport. In this model, we framed identity reformation through the three phases of rites of passage; a process that starts with athletes losing a salient athletic identity upon retiring from their elite-level athletic career that provokes an identity crisis. As athletes experience an identity crisis and move on from this separation phase, they enter the transition rites, navigating through no man’s land and being in limbo. Feeling in between their former elite athlete identity and future identity post-sport life, athletes must cope with emotional turmoil and make psychosocial adjustments before being able to progress to a new meaningful and synthesized identity. In this self-reformation model, we contended that positive identity reformation is accomplished by undergoing a temporary identity moratorium status during the liminal phase and eventually reincorporating into identity achievement post-liminal stage.

However, due to psychological challenges and identity work difficulties of the in between phase, elite athletes in transition may encounter a few roadblocks on their path to identity growth post-sport life. Although identity moratorium serves as a precursor to a progressive structural transformation in the identity formation process, this transitory status does not always lead to identity growth (Côté and Schwartz, 2002; Meeus et al., 2010). Given that psychosocial development and liminal experiences occurring later on in life would be affected by the resolutions of previous identity crises (Kroger, 2015; Ibarra and Obodaru, 2016), it is critical that the identity crisis experienced during the life after sport transition results in a positive identity development post-liminal stage. The importance of this developmental opportunity for athletes’ functioning during adulthood warranted the need to identify key conditions, triggers, and processes that would foster identity growth during this liminal phase.

Experiencing transition conditions that afford greater degrees of freedom, undergoing transition triggers of confusion and ambivalence, and avoiding to prematurely commit to conferred identity options, while exploring a few ones in-depth rather than remaining trapped in an incessant exploration in-breadth, are all favorable aspects that buttress the identity growth paradox of the life after sport transition. As Waterman (2015, p. 312) puts it somewhat poetically, “it appears that going through the valley of distress is the route to the peaks of self-understanding and well-being.” In the search for new directions in life after elite sport, athletes who embrace the fertile emptiness of liminality are likely to capitalize on this liminal opportunity to grow, which demonstrates that this identity growth paradox experienced during the transition serves as a catalyst toward identity achievement.

As suggested, greater freedom and flexibility experienced during the life after sport transition enable elite athletes to be more innovative, agentic, and idiosyncratic in constructing their next self. While taking advantage of this enhanced autonomy and diminished normative societal structure during this liminal experience would facilitate identity growth, these conditions may render feelings of confusion and ambivalence uncomfortable to endure. However, the awakening of these feelings can act as catalysts for identity growth by inciting elite athletes to invest in identity work and engage in existential inquiry to reach a decision and establish a constructed set of core values, beliefs, and goals that will endow direction and meaning in their life after sport.

Finally, critical processes to forge an achieved identity for once an athlete’s sport career ends mainly consist of maintaining exploratory work until enough information and experiences have been gained to make a decision to establish firm identity commitments and resolve the identity crisis. Thus, elite athletes must not prematurely commit to conferred alternatives to avoid experiencing challenging identity search. While they are at risk of shortening the exploratory process to seemingly cope with discomfort and distress by hastily settling into a conferred sense of self, they are also vulnerable to fall into perpetual ruminations, which may paralyze their abilities to make decisions. The path to growing into an achieved identity post-sport life involves making a trade-off between ending hastily identity work processes for avoidance of discomfort and lingering in incessant exploratory process for fear of making the wrong choice.

While a conforming, avoiding, or ruminating approach would not be recommended to establish constructed identity commitments, the source of developmental problems appears to emerge from considering broadly, various identity alternatives rather than implementing and evaluating deeply, fewer options. Therefore, support and oversight must be provided to elite athletes in the early stages of identity search in order to ensure that initial identity work eventually leads them to narrow down their options to lessen the risk of stagnating or even regressing in addressing identity concerns related to their transition to life after elite sport. Elite athletes must be encouraged to persevere in this challenging search for a meaningful identity and delay commitments for as long as it is necessary to achieve identity growth in spite of experiencing uncomfortable feelings of void, ambiguity, and uncertainty during this liminal phase. Certain identity elements of their former athletic self must be negotiated, retained, integrated, and adapted to the new identity. Therefore, reforming into an achieved identity for life after elite sport would corroborate the successful navigation of the transition rites and completion of the rites of passage, as elite athletes evolved into a synthesized sense of self by cementing, through a negotiated adaptation pathway, constructed identity commitments that will provide new beginnings and meaningful directions to their life after elite sport.

## Conclusion

Given that theory building with regards to athletes’ identity reformation upon retirement from elite sport has received only scant attention, in this study, we drew upon conceptual integrations of liminality and identity development to build a model of self-reformation that advances the presence of identity growth paradox during the transition to life after elite sport. Framing the core process of this challenging transition around liminality during which elite athletes are suspended in between the loss of athletic self and the absence of future directions, we offered a series of propositions that not only depict processes of athletes’ identity development during the transition to life after elite sport, but also demonstrate a path for elite athletes to form an achieved identity for their life after sport.

We hope that this integrative framework not only informs future empirical research on athletes’ identity reformation processes during their transition to life after elite sport, but that it will also stimulate further theoretical expansions on the dynamics of positive identity development during various transitions that trigger liminal experiences throughout the lifespan. Given the developmental and psychosocial challenges related to exploration and identity work, the proposed transition conditions and triggers as well as identity work processes underlying the identity growth paradox experienced during liminality merit further empirical examination, especially in terms of its function as a catalyst toward identity achievement. Providing a mixed approach of qualitative and quantitative evidence collected longitudinally in order to test these propositions is recommended to shed light on the complexities of liminal experiences and related identity growth and reformation.

While liminal experiences can offer a structured but idiosyncratic and rich account of identity development and related psychosocial challenges, the conceptual integrations with the identity status paradigm can indicate individuals’ position in the model by using existing self-reported measures of identity status (Crocetti et al., 2008; Luyckx et al., 2008). This paradigm can also provide systematic and directly testable relationships underlying this model of self-reformation. In addition, the abundant literature on narrative identity and its commonly assessed variables such as meaning-making, exploratory processing, agency, and redemption can be applied in this line of inquiry to bring the nuance and clarity needed to our understanding of identity growth and self-reformation (Adler et al., 2017; Kerr et al., 2019).

While, we believe that the transition to life after elite sport provides a unique and fruitful context to study these critical theoretical integrations, we also aimed to inform practice by offering a plausible model that could be used to support elite athletes in need when navigating challenging transitions upon retirement from sport. This self-reformation model can in fact guide the development of educational and clinical interventions that are specifically targeted to athletes with similar identity functioning in order to anticipate transition challenges, promote their identity development and in turn enhance their quality of life after elite sport.

## Author Contributions

Both the authors listed have made a substantial, direct and intellectual contribution to the work, and approved it for publication.

### Conflict of Interest

The authors declare that the research was conducted in the absence of any commercial or financial relationships that could be construed as a potential conflict of interest.

## References

[ref1] Adler J. A. Dunlop W. L. Fivush R. Lilgendahl J. P. Lodi-Smith J. McAdams D. P.. (2017). Research methods for studying narrative identity: a primer. Soc. Psychol. Personal. Sci. 8, 519–527. 10.1177/1948550617698202

[ref2] Ashforth B. E. (2001). Role Transitions in Organizational Life: An Identity-Based Perspective. Mahwah, NJ: Lawrence Erlbaum Associates.

[ref3] Ashforth B. E. Rogers K. M. Pratt M. G. Pradies C. (2014). Ambivalence in organizations: a multilevel approach. Organ. Sci. 25, 1443–1478. 10.1287/orsc.2014.0909

[ref4] Beech N. (2011). Liminality and the practices of identity reconstruction. Hum. Relat. 64, 285–302. 10.1177/0018726710371235

[ref5] Bowers M. T. (2011). Playing video games as a supplement to identity: insights on former college athlete transitions. J. Issues Intercoll. Athl. 4, 289–308.

[ref6] Brewer B. W. Petitpas A. J. (2017). Athletic identity foreclosure. Curr. Opin. Psychol. 16, 118–112. 10.1016/j.copsyc.2017.05.004, PMID: 28813333

[ref7] Brewer B. W. Van Raalte J. L. Petitpas A. J. (2000). “Self-identity Issues in Sport Career Transitions,” in Career Transitions in Sport: International Perspectives. eds. Lavallee D. Wylleman P. (Morgantown WV: Fitness Information Technology), 29–43.

[ref8] Bridges W. (1980). Transitions: Making Sense of Life’s Changes. Reading, MA: Addision-Wesley.

[ref9] Butt J. Molnar G. (2009). Involuntary career termination in sport: a case study of the process of structurally induced failure. Sport Soc. 12, 240–257. 10.1080/17430430802591027

[ref10] Cavallerio F. Wadey R. Wagstaff C. R. D. (2017). Adjusting to retirement from sport: narratives of former competitive rhythmic gymnasts. Qual. Res. Sport Exerc. Health 9, 533–545. 10.1080/2159676X.2017.1335651

[ref11] Chalip L. (2008). Towards social leverage of sport events. J. Sport Tour. 11, 109–127. 10.1080/14775080601155126

[ref12] Coakley J. J. (1983). Leaving competitive sport. Retirement or rebirth? Quest 35, 1–11. 10.1080/00336297.1983.10483777

[ref13] Conroy S. A. O’Leary-Kelly A. M. (2014). Letting go and moving on: work-related identity loss and recovery. Acad. Manag. Rev. 39, 67–87. 10.5465/amr.2011.0396

[ref14] Côté J. E. (2006). “Emerging Adulthood as an Institutionalized Moratorium: Risks and Benefits to Identity Formation,” in Emerging Adults in America: Coming of Age in the 21st Century. eds. Arnett J. J. Tanner J. L. (Washington DC: APA), 85–116.

[ref15] Côté J. E. (2015). “Identity Formation Research From a Critical Perspective: Is a Social Science Developing?,” in The Oxford Handbook of Identity Development. eds. McLean K. C. Syed M. (New York NY: Oxford University Press), 527–538.

[ref16] Côté J. E. Schwartz S. J. (2002). Comparing psychological and sociological approaches to identity: identity status, identity capital, and the individualization process. J. Adolesc. 25, 571–586. 10.1006/jado.2002.0511, PMID: 12490176

[ref17] Crocetti E. Meeus W. (2015). “The Identity Statuses: Strengths of a Person-Centered Approach,” in The Oxford Handbook of Identity Development. eds. McLean K. C. Syed M. (New York NY: Oxford University Press), 97–114.

[ref18] Crocetti E. Rubini M. Luyckx K. Meeus W. (2008). Identity formation in early and middle adolescents from various ethnic groups: from three dimensions to five statuses. J. Youth Adolesc. 37, 983–996. 10.1007/s10964-007-9222-2

[ref19] Cummins P. O’Boyle I. (2015). Psychosocial factors involved in transitions from college to postcollege careers for male NCAA Division-1 basketball players. J. Career Dev. 42, 33–47. 10.1177/0894845314532713

[ref20] Douglas K. Carless D. (2009). Abandoning the performance narrative: two women’s stories of transition from professional sport. J. Appl. Sport Psychol. 21, 213–230. 10.1080/10413200902795109

[ref21] Drahota J. A. T. Eitzen D. S. (1998). The role exit of professional athletes. Sociol. Sport J. 15, 263–278. 10.1123/ssj.15.3.263

[ref22] Dutton J. Roberts L. M. Bednar J. (2010). Pathways for positive identity construction at work: four types of positive identity and the building of social resources. Acad. Manag. Rev. 35, 265–293. 10.5465/AMR.2010.48463334

[ref23] Ebaugh H. R. F. (1988). Becoming an Ex: The Process of Role Exit. Chicago, IL: University of Chicago Press.

[ref24] Erikson E. H. (1959). “Identity and the Life Cycle: Selected Papers” In Psychological Issues. New York: International Universities Press.

[ref25] Erikson E. H. (1963). Childhood and Society. 2nd Edn. New York: W. W. Norton. & Company.

[ref26] Erikson E. H. (1968). Identity: Youth and Crisis. New York, NY: W. W. Norton & Company.

[ref27] Feiler B. (2020). Life is in the Transitions: Mastering Change at Any Age. New York, NY: Penguin Press.

[ref28] Gairdner S. E. (2015). The Making and Unmaking of Elite Athletes: The Body Informed Transition Out of Sport. [Unpublished Doctoral Dissertation]. University of Toronto.

[ref29] Gordon L. Rees C. E. Jindal-Snape D. (2020). Doctors’ identity transitions: choosing to occupy a state of ‘betwixt and between’. Med. Educ. 54, 1006–1018. 10.1111/medu.14219, PMID: 32402133

[ref30] Green B. C. Chalip L. (1998). Sport tourism as the celebration of subculture. Ann. Tour. Res. 25, 275–291. 10.1016/S0160-7383(97)00073-X

[ref31] Grove R. Lavallee D. Gordon S. (1997). Coping with retirement from sport: the influence of athletic identity. J. Appl. Sport Psychol. 9, 191–203. 10.1080/10413209708406481

[ref32] Grove R. Lavallee D. Gordon S. Harvey J. H. (1998). Account-making: a model for understanding and resolving distressful reactions to retirement from sport. Sport Psychol. 12, 52–67. 10.1123/tsp.12.1.52

[ref33] Hennekam S. Bennett D. (2016). Involuntary career transition and identity within the artist population. Pers. Rev. 45, 1114–1131. 10.1108/PR-01-2015-0020

[ref34] Holding A. Fortin J. A. Carpentier J. Hope N. Koestner R. (2020). Letting go of gold: examining the role of autonomy in elite athletes’ disengagement from their athletic careers and well-being in retirement. J. Clin. Sport Psychol. 14, 88–108. 10.1123/jcsp.2018-0029

[ref35] Holstein J. A. Jones R. S. Koonce G. E. (2015). Is There Life After Football? Surviving the NFL. New York, NY: New York University Press.

[ref36] Ibarra H. (1999). Provisional selves: experimenting with image and identity in professional adaptation. Adm. Sci. Q. 44, 764–791. 10.2307/2667055

[ref37] Ibarra H. Barbulescu R. (2017). Identity as narrative: prevalence, effectiveness, and consequences of narrative identity work in macro work role transitions. Acad. Manag. Rev. 35, 135–154. 10.5465/amr.35.1.zok135

[ref38] Ibarra H. Obodaru O. (2016). Betwixt and between identities: liminal experience in contemporary careers. Res. Organ. Behav. 36, 47–64. 10.1016/j.riob.2016.11.003

[ref40] Kerr G. Dacyshyn A. (2000). The retirement experiences of elite, female gymnasts. J. Appl. Sport Psychol. 12, 115–133. 10.1080/10413200008404218

[ref41] Kerr D. J. R. Deane F. P. Crowe T. P. (2019). Narrative identity reconstruction as adaptive growth during mental health recovery: a narrative coaching boardgame approach. Front. Psychol. 10:994. 10.3389/fpsyg.2019.00994, PMID: 31133932 PMC6517514

[ref42] Knights S. Sherry E. Ruddock-Hudson M. (2016). Investigating elite end-of-athletic-career transition: a systematic review. J. Appl. Sport Psychol. 28, 291–308. 10.1080/10413200.2015.1128992

[ref43] Kroger J. (1996). Identity, regression, and development. J. Adolesc. 19, 203–222. 10.1006/jado.1996.0020, PMID: 9245278

[ref82] Kroger J. (2015). “Identity Development Through Adulthood: The Move Toward “Wholeness”” In The Oxford Handbook of Identity Development. eds. McLean K. C. Syed M. (New York: Oxford University Press), 65–80.

[ref44] Kroger J. Marcia J. E. (2011). “The Identity Statuses: Origins, Meanings, and Interpretations,” in Handbook of Identity Theory and Research. eds. Schwartz S. J. Luyckx K. Vignoles V. L. (New York: Springer), 31–53.

[ref45] Kunnen S. E. Metz M. (2015). “Commitment and Exploration: the Need for a Developmental Approach,” in The Oxford Handbook of Identity Development. eds. McLean K. C. Syed M. (New York NY: Oxford University Press), 115–131.

[ref46] Lally P. (2007). Identity and athletic retirement: a prospective study. Psychol. Sport Exerc. 8, 85–99. 10.1016/j.psychsport.2006.03.003

[ref47] Lavallee D. Gordon S. Grove J. R. (1997). Retirement from sport and the loss of athletic identity. J. Pers. Interpers. Loss 2, 129–147. 10.1080/10811449708414411

[ref48] Lavallee D. Nesti M. Borkoles E. Cockerill I. Edge A. (2000). “Intervention Strategies for Athletes in Transition,” in Career Transitions in Sport: International Perspectives. eds. Lavallee D. Wylleman P. (Morgantwon, WV: Fitness Information Technology), 111–130.

[ref49] Lavallee D. Robinson H. K. (2007). In pursuit of an identity: a qualitative exploration on retirement from women’s artistic gymnastics. Psychol. Sport Exerc. 8, 119–141. 10.1016/j.psychsport.2006.05.003

[ref50] Lupo C. Brustio P. R. Valentic E. Kiendl D. Wenzel R. Stockinger W.. (2018). The use of focus group interviews to define the perceived importance of competencies related to the entrepreneurship as starting point for a new career in European athletes: an AtLETyC study. Sport Sci. Health 14, 9–17. 10.1007/s11332-017-0385-2

[ref51] Luyckx K. Schwartz S. J. Berzonsky M. D. Soenens B. Vansteenkiste M. Smits I.. (2008). Capturing ruminative exploration: extending the four-dimensional model of identity formation in late adolescence. J. Res. Pers. 42, 58–82. 10.1016/j.jrp.2007.04.004

[ref52] Lyons L. K. Dorsch T. E. Bell L. F. Mason L. G. (2018). Renegotiating identity: a phenomenological investigation of the college transition for former high school athletes no longer engaged in varsity competition. Identity 18, 18–33. 10.1080/15283488.2017.1410156

[ref53] Maitlis S. (2009). “Who Am I now? Sensemaking and Identity in Posttraumatic Growth,” in Exploring Positive Identities and Organizations: Building a Theoretical and Research Foundation. eds. Roberts L. M. Dutton J. E. (New York: Routledge/Taylor and Francis), 47–76.

[ref54] Marcia J. E. (1966). Development and validation of ego-identity status. J. Pers. Soc. Psychol. 3, 551–558. 10.1037/h0023281, PMID: 5939604

[ref55] Marcia J. E. (1993). “The Status of the Statuses: Research Review,” in Identity: A Handbook for Psychosocial Research. eds. Marcia J. E. Waterman A. S. Matteson D. R. Archer S. L. Orlofsky J. L. (New York: Springer-Verlag), 22–41.

[ref56] Marcia J. E. Waterman A. S. Matteson D. R. Archer S. L. Oflofsky J. L. (1993). Ego Identity: A Handbook for Psychological Research. New York: Springer-Verlag.

[ref57] Mateu P. Ingles E. Torregrossa M. Rodrigues Marques R. F. Stambulova N. Vilanova A. (2020). Living life through sport: the transition of elite Spanish student-athletes to a university degree in physical activity and sports sciences. Front. Psychol. 11:1367. 10.3389/fpsyg.2020.01367, PMID: 32655454 PMC7325594

[ref58] McCabe S. (2006). “The Making of Community Identity Through Historic Festive Practice: the Case of Ashbourne Royal Shrovetide Football,” in Festivals, Tourism, and Social Change. eds. Pickard D. Robinson M. (Clevedon, UK: Channel View Publications), 99–118.

[ref59] Meeus W. J. van de Schoot R. Keijsers L. Schwartz S. J. Branje S. (2010). On the progression and stability of adolescent identity formation: a five-wave longitudinal study in early-to-middle and middle-to-late adolescence. Child Dev. 81, 1565–1581. 10.1111/j.1467-8624.2010.01492.x, PMID: 20840241

[ref60] Park S. Lavallee D. Tod D. (2013). Athletes’ career transition out of sport: a systematic review. Int. Rev. Sport Exerc. Psychol. 6, 22–53. 10.1080/1750984X.2012.687053

[ref61] Pratt M. G. Pradies C. (2011). “Just a Good Place to Visit? Exploring Positive Responses to Psychological Ambivalence” In The Oxford Handbook of Positive Organizational Scholarship. eds. Cameron K. S. Spreitzer G. M. (New York: Oxford University Press), 924–937.

[ref62] Rowe S. (2008). “Chapter Six Modern Sports: Liminal Ritual or Liminoid Leisure,” in Victor Turner and Contemporary Cultural Performance. ed. John G. S. (Brooklyn, NY: Berghaln Books, Inc.), 127–148.

[ref63] Schwartz S. J. (2007). The structure of identity consolidation: multiple correlated constructs or one superordinate construct? Identity 7, 27–49. 10.1080/15283480701319583

[ref64] Schwartz S. J. Beyers W. Luyckx K. Soenens B. Zamboanga B. L. Forthun L. F.. (2011). Examining the light and dark sides of emerging adults’ identity: a study of identity status differences in positive and negative psychosocial functioning. J. Youth Adolesc. 40, 839–859. 10.1007/s10964-010-9606-6, PMID: 21088875 PMC7870419

[ref65] Schwartz S. J. Zamboanga B. L. Luyckx K. Meca A. Ritchie R. A. (2013). Identity in emerging adulthood: reviewing the field and looking forward. Emerg. Adulthood 1, 96–113. 10.1177/2167696813479781

[ref66] Sinclair D. Orlick T. (1993). Positive transitions from high performance sport. Sport Psychol. 7, 138–150. 10.1123/tsp.7.2.138

[ref67] Soderlund J. Borg E. (2017). Liminality in management and organization studies: process, position and place. Int. J. Manag. Rev. 20, 880–902. 10.1111/ijmr.12168

[ref68] Stambulova N. (2003). “Symptoms of a Crisis-Transition: a Grounded Theory Study,” in SIPF Yearbook 2003. ed. Hassmen N. (Orebro, Sweden: University Press), 97–109.

[ref69] Stephan Y. Bilard J. Ninot G. Delignieres D. (2003). Repercussions of transition out of elite sport on subjective well-being: a one-year study. J. Appl. Sport Psychol. 15, 354–371. 10.1080/714044202

[ref70] Stier J. (2007). Game, name, and fame: afterwards, will I still be the same? Int. Rev. Sociol. Sport 42, 99–111. 10.1177/1012690207081830

[ref71] Taylor J. Ogilvie B. C. (2001). “Career Termination Among Athletes,” in Handbook of Sport Psychology. 2nd Edn. eds. Singer R. Hausenblas H. Janelle C. (New York, NY: John Wiley & Sons, Inc.), 672–694.

[ref72] Turner V. W. (1967). “Betwixt and Between: The Liminal Period in Rites de Passage” In The Forest of Symbols: Aspects of Ndembu Ritual. Ithaca: Cornell University Press, 93–111.

[ref74] van Gennep A. (1960). (First published in 1908.) The Rites of Passage. (Translated by Vizedom M. B. Caffee G. L.) Chicago: University of Chicago Press.

[ref75] Warriner K. Lavallee D. (2008). The retirement experiences of elite gymnasts: self-identity and the physical self. J. Appl. Sport Psychol. 20, 301–317. 10.1080/10413200801998564

[ref76] Waterman A. S. (2015). What does it mean to engage in identity exploration and to hold identity commitments? A methodological critique of multidimensional measures for the study of identity processes. Identity 15, 309–349. 10.1080/15283488.2015.1089403

[ref77] Wendling E. Sagas M. (2019). Career Identity Formation in the Transition to Life After College Sport: An Assessment of Career Identity Status and Psychosocial Functioning. Available at: https://ncaaorg.s3.amazonaws.com/research/grants/gsrg/RES_GSRGSummariesFindings.pdf (Accessed September 23, 2019).

[ref78] Wendling E. Sagas M. (2020). An application of the social cognitive career theory model of career self-management to college athletes’ career planning for life after sport. Front. Psychol. 11:9. 10.3389/fpsyg.2020.00009, PMID: 32038437 PMC6993061

[ref79] Willard V. C. Lavallee D. (2016). Retirement experiences of elite ballet dancers: impact of self-identity and social support. Sport Exerc. Perform. Psychol. 5, 266–279. 10.1037/spy0000057

[ref80] Wittman S. (2019). Lingering identities. Acad. Manag. Rev. 44, 724–745. 10.5465/amr.2015.0090

[ref81] Wylleman P. (2019). “A Developmental and Holistic Perspective on Transiting Out of Elite Sport,” in APA Handbook of Sport and Exercise Psychology. Sport Psychology. *Vol*. 1. eds. Anshel M. H. Petrie T. A. Steinfeldt J. A. (Washington, DC: American Psychological Association), 201–216.

